# Ochratoxins in Wines: A Review of Their Occurrence in the Last Decade, Toxicity, and Exposure Risk in Humans

**DOI:** 10.3390/toxins13070478

**Published:** 2021-07-10

**Authors:** Bryan Ortiz-Villeda, Olga Lobos, Kateryn Aguilar-Zuniga, Verónica Carrasco-Sánchez

**Affiliations:** Department of Microbiology, Faculty of Health Sciences, Talca University, Talca 3460000, Chile; bryan.ortiz@utalca.cl (B.O.-V.); olobos@utalca.cl (O.L.); kateryn.aguilar@utalca.cl (K.A.-Z.)

**Keywords:** mycotoxins, ochratoxins, wines

## Abstract

Ochratoxins (OTs) are mycotoxins frequently found in wines, and their contamination can occur during any stage of the winemaking process. Ochratoxin A (OTA) has been the most widely reported and the only one whose concentrations are legislated in this beverage. However, ochratoxin B, ochratoxin A methyl ester, ochratoxin B methyl ester, ochratoxin A ethyl ester, ochratoxin B ethyl ester, ochratoxin α, ochratoxin β, OTα methyl ester, OTA ethyl amide, and OTA glucose ester have also been reported in wines. Thus, detecting only OTA would lead to the underestimation of ochratoxin levels, which is a risk to human health. Considering the threat represented by the presence of ochratoxins in wines and the long-term health problems that they can cause in wine drinkers, this paper aims to review reports of the last 10 years regarding the presence of different ochratoxins in wines and how the winemaking process influences the degree of contamination, mainly by OTA. Additionally, toxicity from human exposure due to the consumption of contaminated wines is addressed.

## 1. Introduction

**Figure 1:**
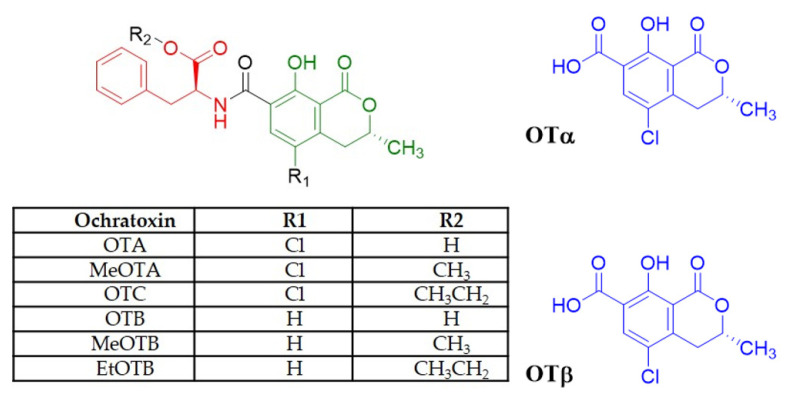
Ochratoxins structure. The red color represents the phenylalanine moiety deriving from the shikimic acid pathway. The green color represents the isocoumarin nucleus. OTA: ochratoxin A; MeOTA: ochratoxin A methyl ester; OTC: ochratoxin C; OTB: ochratoxin B; MeOTB: ochratoxin B methyl ester; EtOTB: ochratoxin B ethyl ester, OTα, and OTβ.

Ochratoxins (OTs) are a group of mycotoxins produced by different *Penicillium* and *Aspergillus* molds that contaminate crops in the field [1]. These toxins are derivatives of an isocoumarin moiety linked to phenylalanine by an amide bond (Figure 1) [2].

In wines, OTA is the most studied mycotoxin, and the European Commission (by regulation 1881/2006) established as the maximum tolerable level in wines destined for human consumption a concentration of 2 µg kg^−1^ [3]. OTA contamination can occur during any stage of the winemaking process. Contamination can be produced from the early stages of the colonization of mycotoxigenic fungi in grapes to the final steps in the wine packaging process. However, the primary contamination of the finished product comes from the carryover of mycotoxins from grapes [4,5]. In addition, the winemaking process strongly influences the OTA content, since higher concentrations have been reported in red wines than in rosé and white wines in general [6]. 

Ochratoxins other than OTA have been reported in wines [7], but because they present different polarity and solubility, their detection and quantification using conventional analytical methods becomes difficult, leading to underestimations of total mycotoxin levels [7,8]. So far, the other ochratoxins present in wines have been identified as ochratoxin B (OTB), ochratoxin A methyl ester (MeOTA), ochratoxin B methyl ester (MeOTB), ochratoxin A ethyl ester (OTC), ochratoxin B ethyl ester (EtOTB), ochratoxin α (OTα, an isocoumarin derivative of OTA) ochratoxin β (OTβ, a dechlorinated analog of OTα), OTα methyl ester, OTA ethyl amide, and OTA glucose ester [9,10,11]. Although they are not regulated in wine, their presence is undoubtedly a risk to human health.

The contamination of wines with ochratoxins should raise a tremendous public health alert worldwide for frequent drinkers, since these toxins can cause acute to chronic poisoning. The latter is associated with the cumulative effect of these toxins in various organs when they are ingested constantly over a long time and can cause the occurrence of chronic complications such as cancer, immunological alterations, nephropathies, neurotoxicity, and hepatotoxicity, among others [12].

The presence of OTA in wines has been reviewed by several authors [6,13,14]; however, most of these publications covered reports only until 2012. Considering the risk of contamination by ochratoxins and the long-term health problems they can cause, this review aims to analyze the presence of ochratoxins in wines reported in the last 10 years, explore how the winemaking process influences the degree of contamination, and examine the associated toxicity in terms of the risk of human exposure from the consumption of contaminated wines.

## 2. Occurrence of Ochratoxins in Wines

### 2.1. Ochratoxin A

OTA structurally consists of a *p*-chlorophenolic group linked to a dihydroisocoumarin fragment linked by an amide bond to an L-phenylalanine (Figure 1) [15].

According to some research, OTA contamination occurs when grapes are still on the vine, during the ripening period when fungal infections are more active [16,17]. *Aspergillus carbonarius, A. ochraceus, A. niger, Penicillium verrucosum,* and *P. nordicum* are the main fungal species responsible for producing this toxin in grapes [18], with *Aspergillus* species being more frequent [19]. The grape harvesting process is critical in terms of contamination with mycotoxigenic fungi, especially if there is a lack of cleanliness of the tools and containers used. Similarly, contamination of grapes can also occur during transport to the winery, during storage, or because of careless handling by staff, since the skin of these fruits is thin and can be easily injured, allowing colonization and development of mycotoxigenic fungal species [20]. 

In the crushing step, grapes are broken to extract the juice. When grapes contain OTA (in their pulp and/or skin), the toxin will be transferred to the must [6].

For red wines, the maceration process is then carried out. During this stage, there can be increased OTA content (about 20%) [21] because of the long contact between grape skins and must, which favors the solubility and diffusion of this mycotoxin from contaminated skins [22]. On the other hand, the absence of maceration for white and rosé wines seems to be a critical factor contributing to low OTA levels in these wines [14].

After maceration for red wines and crushing for white and rosé wines, alcoholic fermentation occurs, a process by which the sugars present in the grapes, thanks to the metabolism of yeasts, are converted into alcohol. Although mycotoxigenic fungi can accompany the grapes from the time they are on the vine, the fermentation process inhibits their growth [23]. It has even been shown that living spores of *A. carbonarius* isolated during alcoholic fermentation cannot produce OTA [24]. Along with this, at the end of the alcoholic fermentation process, OTA levels decrease by 35 to 70% [14].

OTA adsorption can be carried by yeast cell walls (composed of mannoproteins and β-glucans) [25]. It was shown that chitin, β-glucan, and their hydrolysates could remove 64 to 74% of OTA from contaminated wine [26]. A similar process occurs during malolactic fermentation, which involves lactic acid bacteria (LAB) [27]. Some authors have reported the ability of LABs to decrease OTA concentrations between 10 and 43% during the winemaking process [28]. OTA removal for LAB would be in the form of adsorption to cellular components such as exopolysaccharides and peptidoglycans [21,28].

A 54% reduction in OTA content has been seen in wines stored for 5 months [6], and OTA concentrations in red wine stored at room temperature for 55 days and 80 days showed decreases of 8 and 10%, respectively [29]. The OTA reduction in wine after storage and drainage is due to the precipitation of solid particles from the remains of pulp, skin, and yeast and dead bacteria, which form the lees [6]. This could be due to the wine sedimentation process, which contributes to further reduction of OTA content [13,30].

After clarification with gelatin in the macro-vinification process, there was evidence of a decrease in toxin content by around 58%. On the other hand, using a mixture of gelatin and bentonite, the concentration of OTA does not decrease significantly [6]. Other clarifying agents, such as oenological carbon and bentonite, have been shown to reduce the OTA concentration in wine [31,32]. However, the fining agent concentration, the chemical nature of the wine components, the OTA concentration, and the adsorption capacity of other elements present in the wine must be considered.

Finally, throughout the winemaking process, evidence shows that OTA levels can be decreased. Recently, a new scenario was raised, in which a third of the reduction may be related to the degradation of OTA or transformation into modified mycotoxins during the fermentation process [8].

The presence of OTA in wine was first reported in 1996 in Switzerland [33]. Since that first report, this mycotoxin has been described in different wines on several countries worldwide. Table 1 lists the wine reports from 2012. Among the reports that most attract attention is one in which 100 samples of wine produced in Portugal, Spain, and Italy were analyzed. The interesting thing about this study is that the year of production varied from 1984 to 2017, and interestingly, the contaminated samples were from wine made in recent years, two red wines in Portugal in 2016, one red wine in Lisbon in 2016, and one in Italy in 2015. The contaminated white wine that was sampled was produced in Italy in 2016 [34].

Another work that draws attention is a recent study that analyzed 113 bottled wines from the 2011 to 2016 vintages. Of the total wines evaluated, 64% of red wines had at least traces of OTA, while the percentages of white and rosé wines were considerably lower (42.6 and 36.4%, respectively). Regarding the harvest year, the occurrence of OTA varied markedly. The samples with the highest levels of contamination were from the 2014 and 2015 harvests (71.0 and 86.7%, respectively) [35].

It is essential to have these data because of the increased OTA in wines after 2014 [34,35], which, according to some researchers, may be due to the consequences of climate change.

### 2.2. Other Ochratoxins

The alcoholic and acidic nature of wine allows carboxylic acid transformation to an ethanolic ester in OTA [33]. Furthermore, the acidic conditions of the matrix may favor ionization of the amino group of the OTA molecule. Along with this, acids can favor esterification reactions (addition of methyl and ethyl groups). Furthermore, during the alcoholic fermentation process, the enzymes produced by the yeasts present can modify OTA. *Saccharomyces cerevisiae* has been shown to produce glucosidases, pectinases, and xylanases that can act on the hydrolysis of OTA [8].

The first report of co-occurrence of OTA with OTC in wines was in 1996, estimating that the OTC concentration was approximately 10% of the OTA concentration (Table 2) [33]. Subsequently, in 2010, it was reported that 100% of red wines purchased from Spain presented co-occurring OTA and OTB although at low levels, and none of them exceeded the maximum level of OTA permitted by legislation. Additionally, it was reported that 60% of the samples presented three ochratoxins (eight showed OTA, OTB, and OTC, and four, OTA, OTB, and MeOTA) [9].

OTA, OTB, MeOTA, MeOTB, OTC, and EtOTB were reported in 51 red wines from Spain, corroborating previously published data, with levels of OTA and OTB detected in all wines. Among the samples, 71% showed the presence of OTC, and in 18%, the six ochratoxins analyzed appeared simultaneously [36]. Another study reported that all wines obtained in the Mediterranean were contaminated with OTB, 89.6% with OTC, 62.5% with MeOTA, 83.3% with MeOTB, and 83% with EtOTB. The co-occurrence of two mycotoxins in 100% and six ochratoxins in 44.8% of the samples was also found. It has been reported that the presence of OTC in wine is mainly due to the hydrolysis of OTA [60]. Therefore, ochratoxin intake from wine can be underestimated when only assessed by OTA analysis.

## 3. Toxicity of Ochratoxins and Risk of Exposure through the Consumption of Contaminated Wine

### 3.1. OTA Toxicity and Biotransformation

For humans, the ingestion of mycotoxins constitutes a danger, as they have been linked to mutagenicity and to estrogenic, gastrointestinal, urogenital, vascular, renal, nervous, and immunosuppressive disorders [61]. Among their most alarming characteristics is their cumulative effect over time, representing a triggering factor in several types of cancer [62,63]. Of all the mycotoxins reported in wines, OTA is currently considered the most relevant. This molecule has been mainly associated with a nephrotoxic effect, as observed in Balkan endemic nephropathy (BEN), caused by the bioaccumulation of OTA in the renal parenchyma [64,65]. Other effects attributed to this mycotoxin are teratogenesis, immunotoxicity, genotoxicity, mutagenicity, and oncogenicity [65].

After being ingested, a minor fraction of OTA is absorbed in the stomach (due to the rapid chyme transit and the thickness of its mucous layer) [66,67,68]. Contrary to what happens in the small intestine, particularly between the duodenum and the jejunum, which is where more significant amounts of OTA are absorbed, because the evacuation of alimentary chyme is slow, the membranes in this anatomical area are more permeable, which facilitates the absorption of this mycotoxin [66,67]. Another variable that is strongly involved in absorption is the pH of this mycotoxin. The p*K*_a_ of OTA ranges from 4.2 to 4.4 (monoanionic form) and 7.2 to 7.4 (dianionic form); absorption of OTA, according to the physiological pH found in the stomach and small intestine (ranging from 1.5 to 2.5 and 6.1 to 7.8, respectively) occurs in its non-anionic and monoanionic forms [65]. It is important to note that when this mycotoxin is absorbed through the small intestine, it can affect the integrity of the walls, causing inflammation and intestinal disorders [68].

When the binding of OTA to albumin occurs, it is brought to different tissues [69]. OTA is metabolized very slowly in humans, with a half-life of more than 30 days [70,71], producing a cumulative effect leading to cell damage in different organs [71]. The main organs and tissues targeted by OTA are the kidneys, liver, skeletal muscle, adipocytes, and brain [65,66].

Due to all of these contraindications, the International Agency for Research on Cancer (IARC) in 1993 classified OTA in group 2B as a possible carcinogen for humans, which means that there is some evidence that it can cause cancer in humans, but so far, it is not conclusive [72]. However, currently, it is considered that there should be a change in this classification, since investigations carried out with experimental animals during the last 20 years confirm that this mycotoxin is carcinogenic, nephrotoxic, and genotoxic and is also associated with gallbladder cancer due to the consumption of contaminated food [73].

**Figure 2:**
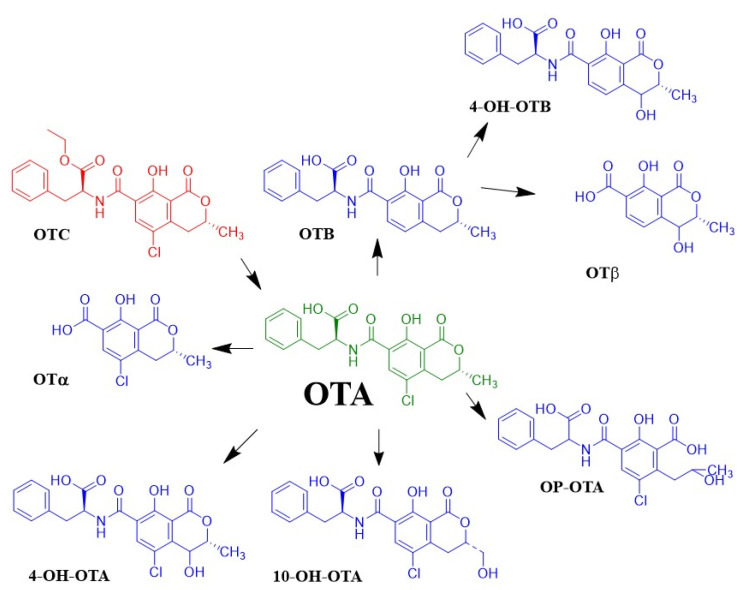
Biotransformation associated with OTA.

When OTA is ingested, it can undergo biotransformations (Figure 2). OTA is hydrolyzed to OTα by the action of proteolytic enzymes and enzymes of bacterial microflora in the intestine. What makes hydrolysis of OTA possible is the opening of the lactone ring that under alkaline conditions results in forming a highly toxic compound called open OTA with lactone (OP-OTA). Likewise, 4-hydroxyochratoxin A (4-OH-OTA) is a product of OTA oxidation, which is a compound with low toxicity, while another less toxic product is 10-hydroxyochratoxin A (10-OH-OTA) [66].

In addition, the formation of other metabolites derived from the biotransformation of OTA has been observed in other species, such as OTB, and the result of the conjugation of OTA with compounds such as sulfate, glucuronide acid, hexose/pentose (hex/pen-OTA), and glutathione, among others. The production of these metabolites is essential to consider because they can interact with other molecules, leading to more toxic molecules. Ochratoxin C is a potentially toxic compound. It has been concluded that ochratoxin C is readily converted to ochratoxin A after either oral or intravenous administration [66,74]

It is also important to note that the total consumption of OTA in the daily diet depends on all the products ingested containing this mycotoxin. Exposure to OTA through food intake can be measured in biological fluids using mycotoxin biomarkers. The OTA concentration in blood is a well-known marker of constant exposure to this toxin due to its long half-life (≈35 days) in the circulatory system [75]. In addition, measuring urinary mycotoxin biomarkers is an effective alternative to measuring exposure to mycotoxins, given that the excretion of biomarkers correlates well with mycotoxin intake [76]. In this regard, a human biomonitoring study performed on the Belgian population revealed that, among 33 mycotoxins analyzed, OTA was one of the most frequently detected in urine samples, indicating a high prevalence of OTA exposure in adults and children in this population associated with food consumption, suggesting the presence of urinary mycotoxin as a valuable biomarker for assessing OTA exposure [77].

### 3.2. Toxicity Associated with OTA Derivates

OTB is less toxic in vivo than OTA, attributed to a lower affinity for plasma proteins, no specific retention in the kidneys, and more extensive metabolization and faster excretion than OTA [78,79]. On the contrary, it has been shown that both toxins can be equally acutely cytotoxic in vitro in exposed LLC-PK1 cells [80], provided that similar amounts are absorbed and bound intracellularly, although several results suggest a molecular mechanism different from chronic toxicity compared to OTA. Furthermore, it can be assumed that the minor structural difference (chlorine in OTA versus hydrogen in OTB), although not responsible for toxicity, may be crucial for differential binding and uptake in cells [81].

OTC has similar acute toxicity in vivo and in vitro compared to OTA; however, it remains to be determined whether the mode of action of OTC and OTA is the same [80]. It has been shown that OTC leads to various immunomodulatory effects in porcine mononuclear cells [82] and the human monocyte/macrophage THP-1 line in concentrations between 10 and 1000 ng mL^−1^ [80].

The derivative 4R-OH-OTA was shown to be equally cytotoxic compared to OTA in HTC cells. Furthermore, both OTA and 4R-OH-OTA inhibit protein synthesis, attributed to their binding on the phenylalanine sites of phenylalanyl-tRNA synthetase, and they possess similar toxic effects on the IgM and IgG response [81].

The derivative 10-OH OTA is non-genotoxic (DNA adduct formation) in bronchial epithelial cells (WI 126 VA) [83,84]. Based on some of the results above, hydroxylation of ochratoxins (particularly 4R-OH-OTA) does not affect their toxicity. In summary, based on the limited data from the above studies, no clear general toxicity ranking can be made; however, OTA seems to be overall the most toxic, followed by OTC, OTB, and OTα [81].

### 3.3. Risk of Exposure to Ochratoxins through the Consumption of Contaminated Wines

In the recent past, wine has been considered the second most important dietary source of OTA after cereals [85]. In women between 18 and 59 years of age, wine is a significant source of OTA consumption [38]. Currently, this represents a contribution of up to 5% of the total intake of this toxin in the elderly population [35].

The European Food Safety Authority (EFSA), based on the renal toxicity that can result from OTA, established a tolerable weekly intake (TWI) level of 120 ng kg^−1^ body weight (bw), which corresponds to a tolerable daily intake (TDI) of 17 ng kg^−1^ bw day^−1^. According to data presented by EFSA, the estimated amount of OTA that an average person could ingest with the consumption of products such as cereals, wine, beer, grapefruit, coffee, pork, and cocoa over the course of a week varies from 15 to 20 ng kg^−1^ bw day^−1^, and in those who consume these foods very frequently, ingestion varies from 40 to 60 ng kg^−1^ per bw week^−1^ [86].

The estimated daily intake (EDI) of mycotoxins through wine consumption can be calculated using the average concentration of mycotoxins in the samples (μg L^−1^), the average consumption of wine per day (L day^−1^), and body weight (kg) [35,50,52]. The EDI of OTA in wines has been evaluated by several researchers, mainly on the European continent (Table 3), and the results so far are in the range of 0.0002 to 4.1 ng kg^−1^ bw day^−1^, indicating that consumption of this mycotoxin is relatively low, considering the TDI proposed by EFSA. It is also important to note that total exposure to OTA in the daily diet will depend on all products consumed that contain it.

Risk assessment for OTA ingestion through contaminated wines has also been carried out by calculating the hazard quotient (HQ) [41,92] and margin of exposure (MOE) [79] values (Table 4).

The hazard quotient, proposed by US Environmental Protection Agency (USEPA), is a tool used to calculate the carcinogenic risk of ingesting a compound that is harmful to health and can represent human exposure through the consumption of such compounds in food and beverages. The HQ of OTA is calculated by dividing the average daily dose (PDD) of mycotoxin in wine by the TDI [41].

The EFSA scientific committee has recommended using the MOE for the risk assessment of substances that are considered genotoxic and carcinogenic and may be present in food at low levels. Briefly, the MOE is a relationship of two factors that can be evaluated in a given population: the dose at which a small but measurable adverse effect is first observed and the level of exposure to a substance considered genotoxic or carcinogenic [86], which is calculated by dividing the lowest benchmark dose (BMDL) by the estimated mycotoxin intake, where BMDL_10_ represents the lower end of the benchmark dose, which indicates an increased risk of cancer by 10% over the control [93,94].

Based on the above, several authors have concluded that wine does not represent a danger to human health as a source of OTA [35,37,41,46,53,54]. Among the justifications for this approach, it is pointed out that despite the presence of OTA in wines, it may be possible that toxic levels of this mycotoxin are somehow offset by the beneficial effects of the derivatives of resveratrol. Additionally, the concentrations found in wine are minimal compared to those found in other food matrices, so the risk of exposure to this mycotoxin through wine consumption is minimal. 

However, these pronouncements are not entirely truthful, as has become evident. Ochratoxin levels in wines may be underestimated, since most studies on the occurrence and risk of exposure were focused on OTA and not its derivatives. Therefore, the presence of other ochratoxins and the lack of concern about detecting them in wine represents a potential risk to human health. 

The presence of these modified metabolites in wines could represent three possible scenarios: (1) a decrease in or elimination of toxicity occurs for the primary molecule [7,8]; (2) these compounds could be as toxic or more toxic than the original molecule due to their structural similarities [8,17]; or (3) when ingested in wine, through human metabolism, these molecules can be reconverted into the original toxin [95]. It should not be ruled out that synergism occurs between modified OTAs and free OTA or other molecules present in this matrix. 

## 4. Conclusions

OTA is a mycotoxin widely studied in wines. The winemaking process is known to influence the presence and concentration of this toxin, since red wines are more contaminated than white and rosé wines. Evidence shows that OTA levels decrease throughout the winemaking process due to loss or degradation. This is a worrying aspect to consider, since the total ochratoxin levels may have been underestimated by not considering the OTA derivates. 

OTA derivatives in wines are OTB, MeOTA, MeOTB, OTC, EtOTB, OTα, OTβ, OTα methyl ester, OTA ethyl amide, and OTA glucose ester of which there is little information on its toxicity. Although analogs analysis is essential, it is somewhat complex, since having different polarities and solubility, making their detection and quantification by conventional analytical methods difficult.

The few studies that analyze OTB in wines show that its prevalence is over 80% when OTA is 100%, with similar concentrations. As for OTC, its prevalence in wines ranges 70%, and its concentration is equivalent to 10% of OTA concentration. 

TDI established by EPSA for OTA is 17 ng kg^−1^ bw day^−1^, and the maximum estimated range of EDI in wines is 4.1 ng kg^−1^ bw day^−1^ in drinkers. The OTA intake through the consumption of contaminated wine corresponds ≈24% of the total daily quota. However, being considered OTB and OTC, it would be estimated that this percentage would exceed 40% of the tolerable daily intake. Although, OTB is less toxic than OTA, and OTC easily converts to OTA after oral administration. Therefore, the underestimation of these derivatives brings with it a worrying scenario for public health. 

The underestimating ochratoxin concentration in wines is accurate, and future studies should focus on evaluating the exposure risk to total ochratoxins and not just OTA. Furthermore, it is essential to carry out a more profound toxicity analysis of OTA derivatives to investigate whether synergism or antagonism between the different ochratoxins and study the cumulative effect on organs and tissues.

## Figures and Tables

**Table 1:** Studies of the occurrence of OTA in wines. Reports from 2012 to 2021.

Sampling Location	Wines	Positive/Total Samples	OTA Range (µg L^−1^)	Method	LOD	Ref.
Portugal	Red, white	5/100	<LOD–1.2	HPLC-FLD	0.08	[34]
Serbia	Red, white, rose	59/113	<LOD–0.13	HPLC-FLD	0.001	[35]
Spain	Red	51/51	0.0005–0.014	HPLC-FLD	0.00032	[36]
Holland	Red	6/280	0.2–0.6	UPL-MS	NR*	[37]
Czech Republic	Red, white	8/24	0.0–0.7	HPLC-FLD	NR*	[38]
Italy	Sweet	29/30	<LOD–1.6	HPLC-FLD	0.01	[39]
Italy	Red, white	55/55	0.08–0.7	UPLC-FLD	0.01	[40]
Poland	-	8/32	0.1–0.5	HPLC-FLD	0.05	[41]
Portugal	Red	4/6	<LOD–0.17	HPLC-FLD	0.017	[42]
Croatia	Red	102/110	<LOD–0.16	HPLC-FLD	0.006	[43]
Greece	-	10/23	3.4–15.6	ELISA	1.0	[44]
Chile	Red, white	34/1188	<LOD–0.4	HPLC-FLD	0.01	[45]
United States of America	Red, white	12/143	0.1– 0.4	UHPLC-MS/MS	0.1	[46]
United States of America	-	6/343	<LOD–0.4	LC-MS/MS	0.1	[47]
Argentina	Red	136/136	<LOD–1	UHPLC-MS/MS	0.02	[48]
United States of America	Red, white, dry, sweet	35/41	0.3–8.6	HPLC-FLD	0.1	[49]
Paraguay	Red	1/4	2.4	ELISA	2.0	[50]
Japan	Red, white	5/27	<0.2–0.4	UHPLC-MS/MS	0.06	[51]
China	Red, white	223/223	<LOD–1	HPLC-FLD	0.01	[52]
Thailand	Red	10/100	0.3–1.7	LC-MS/MS	0.06	[53]
China	-	2/42	1.3	UHPLC-MS/MS	0.1	[54]
Tunisia	Red, white, rose	29/34	0.1–1.5	HPLC-FLD	0.03	[55]
Italy	Red	2/30	2.0	HPLC-FLD	NR*	[56]
Italy	Red	41/57	<LOD–0.7	HPLC-FLD	0.02	[57]
Italy	Red, and white	37/58	<LOD–0.3	HPLC-MS/MS	0.012	[58]
Hungary	Sweet wines (Tokaj)	7/53	<LOD–0.3	HPLC-FLD	0.03	[59]

**Table 2:** Ochratoxin analogues studies in wines.

Wines	Ochratoxin	Positive Wines	Range (µg L^−1^)	Method	LOD (µg L^−1^)	Ref.
Red wines;	OTA	100%	0.001–0.1	HPLC-FLD	0.00016	[9]
*n =* 20	OTB	100%	0.003–0.02		0.00032	
	MeOTA	50%	<LOD–0.001		0.00027	
	OTC	70%	<LOD–0.004		0.00017	
White, rose,	OTA	92.4%	<LOD–0.4	HPLC-FLD	0.003	[33]
and red wines;	OTC	10%	-		NR *	
*n =* 133	OTA + OTC	10%	-			
Red wines;	OTA	100%	0.001–0.01	HPLC-FLD	0.00032	[36]
*n =* 51	OTB	100%	0.003–0.1		0.00016	
	OTC	70.6%	0.0002–0.01		0.00017	
	MeOTA	41.2%	0.0002–0.004		0.00021	
	MeOTB	92.2%	NR*–0.01		NR *	
	EtOTB	43.1%	NR*–0.001		NR *	
Sweet wines;	OTA	96.6%	<LOD–1.6	HPLC-FLD	0.01	[38]
*n =* 30	OTB	83.3%	<LOD–1.2		0.02	
Red wines	OTA	99%	<LOD–0.5	HPLC-FLD	0.00032	[60]
*n =* 96	OTB	100%	0.002–0.1		0.00016	
	MeOTA	62.5%	<LOD–0.1		0.00021	
	OTC	89.6%	<LOD–0.03		0.00017	

* NR: not reported.

**Table 3:** Estimated daily intake (EDI) of OTA for wine consumption.

Country	EDI (ng kg^−1^ bw day^−1^)	Ref.
Switzerland	0.7	[33]
Portugal	0.01	[34]
Serbia	0.004	[35]
Spain	0.01	[36]
Czech Republic	0.01–0.03	[38]
Italy	4.1	[40]
Poland	0.0002	[41]
USA	0.01	[47]
China	0.1–0.2	[52]
Thailand	0.3	[53]
Portugal	2.9–5.4	[87]
Spain	0.01	[88]
France	2	[89]
Greece	3.7	[90]
Italy	0.9–1.4	[91]

**Table 4:** Parameters to determination of risk exposure to OTA due to the consumption of wines.

Parameters	Interpretation of Results	
EDI	<17 ng kg^−1^	The estimated daily intake of OTA should never be greater than the tolerable daily intake that corresponds to 17 ng kg^−1^
HQ	<1	Suggests that carcinogenic effects are unlikely
	>1	Is indicative that the OTA present in the matrix are potential agents that cause adverse health effects
MOE	≥200	Non-neoplastic effects
	≥10,000	Suggests neoplastic effects

Note. EDI: estimated daily intake; HQ: hazard quotient; MOE: margin of exposure.

## Data Availability

Not applicable.
